# Looking towards the future: patient-specific computational modeling to optimize outcomes for transcatheter mitral valve repair

**DOI:** 10.3389/fcvm.2023.1140379

**Published:** 2023-04-24

**Authors:** Paul Wong, Andrew D. Wisneski, Amitoj Sandhu, Zhongjie Wang, Vaikom S. Mahadevan, Tom C. Nguyen, Julius M. Guccione

**Affiliations:** ^1^ School of Medicine, University of California San Francisco, San Francisco, CA, United States; ^2^ Division of Cardiothoracic Surgery, Department of Surgery, University of California San Francisco, San Francisco, CA, United States; ^3^ Division of Cardiology, Department of Medicine, University of California San Francisco, San Francisco, CA, United States

**Keywords:** mitral valve, transcatheter mitral valve repair, computational modeling, finite element, fluid-Structure interaction, patient specific model

## Abstract

Severe mitral valve regurgitation (MR) is a heart valve disease that progresses to end-stage congestive heart failure and death if left untreated. Surgical repair or replacement of the mitral valve (MV) remains the gold standard for treatment of severe MR, with repair techniques aiming to restore the native geometry of the MV. However, patients with extensive co-morbidities may be ineligible for surgical intervention. With the emergence of transcatheter MV repair (TMVR) treatment paradigms for MR will evolve. The longer-term outcomes of TMVR and its effectiveness compared to surgical repair remain unknown given the differing patient eligibility for either treatment at this time. Advances in computational modeling will elucidate answers to these questions, employing techniques such as finite element method and fluid structure interactions. Use of clinical imaging will permit patient-specific MV models to be created with high accuracy and replicate MV pathophysiology. It is anticipated that TMVR technology will gradually expand to treat lower-risk patient groups, thus pre-procedural computational modeling will play a crucial role guiding clinicians towards the optimal intervention. Additionally, concerted efforts to create MV models will establish atlases of pathologies and biomechanics profiles which could delineate which patient populations would best benefit from specific surgical vs. TMVR options. In this review, we describe recent literature on MV computational modeling, its relevance to MV repair techniques, and future directions for translational application of computational modeling for treatment of MR.

## 1. Introduction

Severe mitral regurgitation (MR) is a heart valve disease that features retrograde flow of blood from the left ventricle into the left atrium through the mitral valve, that can be clinically identified as a systolic murmur at the apex of the heart with radiation to the left axilla. MR is regarded as the most common valvular abnormality worldwide, with over 2% of the total population estimated to have the condition (1–4). The etiology of MR can be stratified by primary and secondary causes. Primary MR is attributed to mitral annulus diseases (i.e., mitral annulus calcifications), along with direct structural deformities of the mitral valve leaflets chordae and/or papillary muscles due to degenerative, congenital, or infectious causes (1, 5). Secondary MR, also known as functional or ischemic MR, is generally a byproduct of left ventricular abnormalities or remodeling, leading to changes to the annulus or papillary muscles; however, functional MR can also be attributed to left atrial disease (1). If left untreated, progression of the disease to severe MR can contribute to extensive morbidity and mortality from ensuing atrial fibrillation and congestive heart failure (5).

Due to the potential for heightened morbidity and mortality from untreated mitral regurgitation, surgical management of MR is pivotal for these patients. While medical management options can be pursued for patients with lesser degree MR, the gold standard for treatment of severe MR is surgical MV repair or replacement.

## 2. Current strategies for surgical intervention

While medical therapy alone has limited effect on overall survival for patients with MR, the treatment of choice for MR is surgical intervention (6). Mitral valve surgery can entail repair of the valve, by which the surgeon can apply a variety of techniques such as neochord placement, leaflet resection, and/or annuloplasty ring placement to restore leaflet coaptation to eliminate regurgitation. Within the realm of mitral valve repair, techniques can be subdivided into “respect” vs. “resect” approaches. The “respect” approach entails chordal replacement in which artificial neochord are used to address prolapsed segments of affected leaflets, whereas the “resect” approach involves removal of the diseased leaflet segment (7). Annuloplasty ring placement possesses nuance in strategy as well, with closed and open annuloplasty rings both being used for mitral valve repair. There have been equivocal findings on the comparative functional efficacy and survival between the two rings, but recent studies have suggested similar outcomes between the types of annuloplasty rings (8, 9). Valve replacement remains the other surgical option by which the entire valve is replaced with a mechanical or tissue-based prosthesis. When mitral valve surgery is indicated, repair is generally preferred over replacement when possible and can be achieved in a majority of the cases by an experienced mitral valve surgeon (10). An adequate repair of the mitral valve can last the patient's lifetime, provides optimal hemodynamic function, and does not require the patient to be on anticoagulation. With mitral valve replacement, a tissue prosthesis may be prone to degeneration requiring future reintervention or replacement while mechanical valves require a patient to be on lifelong anticoagulation.

Current surgical strategies for mitral valve repair include the traditional approach of an open surgical repair through either median sternotomy or minimally invasive surgical approaches through mini-thoracotomy. Novel surgical techniques have been increasingly used, such as transapical beating heart mitral valve repair with the NeoChord system (NeoChord, Inc., St. Louis Park, MN, USA). The use of NeoChord implantation has been accompanied with feasibility and safety for patients presenting with severe mitral regurgitation secondary to leaflet prolapse or flail (11, 12). In addition, endovascular options have recently emerged, termed transcatheter mitral valve repair (TMVR). With catheter-based technology, endovascular repair of a mitral valve can be performed with delivery of a clip recreating an edge-to-edge repair to reduce regurgitation. This modern adaptation of the edge-to-edge repair is derived from the Alfieri stitch, which is a technique that features suture placement between the A2 and P2 segments of the mitral valve (13). Thus far, there have only been a few approved devices for this purpose, including the MitraClipTM (Abbott Laboratories, Abbott Park, Illinois, United States) and the Edwards PASCAL Transcatheter Valve Repair System (Edwards Lifesciences, Irvine, California, United States).

Surgical mitral valve repairs have been shown to be durable solutions to MR, as the reported rates of ten-year survival freedom from reoperation varies from 72% to 90% (14). In addition, there is often minimal residual regurgitation following surgical repairs compared to TMVR (15); however, due to the inherent nature of the surgery and need for cardiopulmonary bypass, these repairs may be associated with greater risk of morbidity and mortality compared to endovascular techniques (16, 17).Thus, one advantage of TMVR is to offer a MV repair option for patients at higher surgical risk particularly for older, frail patients with multiple comorbidities (18). It has been demonstrated that patients over the age of 80 that possess significant cardiovascular disease, coagulopathy, and pulmonary disease were shown to have up to a four-fold higher mortality when undergoing surgical MV repairs compared to TMVR (19).

Mechanistically, there are differences between normal mitral valves, surgically repaired ones, and those repaired percutaneously. Surgical mitral valve repairs aim to reinstate the natural geometry of the mitral valve, whereas TMVRs are non-anatomical solutions to MR, as the technique features the implementation of clips that pinch the edge of the mitral valve leaflets to restore proper coaptation. The mechanical effects of MitraClip™ repair have been studied and increases in both end-diastolic and end-systolic mitral leaflet stress have been demonstrated following TMVR (20). These increases in mitral leaflet stress and procedure-related strain in the sub-valvular myocardium have been theorized to contribute to a predisposition for recurrent MR following TMVR.

While patients that are suitable surgical candidates should receive surgical MV repair and high-risk patients can be offered TMVR, the question of how to address a gray zone of intermediate-risk patients is a topic of ongoing study. A meta-analysis by Oh et al. details comparative outcomes between TMVR with MitraClip™ and surgery for MR, highlighting that TMVR provides a similar safety profile at the expense of increased residual MR and higher reoperation rates when compared to surgery (15). Furthermore, a subset analysis of adjusted hazard ratios from studies that included randomized patient recruitment or propensity-match scoring analysis demonstrated a nonsignificant increase in overall mortality for patients that received MitraClip™ compared to surgical MV repair. Patients that received TMVR possessed greater extent of co-morbidities, thus to achieve nonsignificant increase in mortality speaks to the relative safety advantage of TMVR for higher risk patients.

Several ongoing clinical trials are being undertaken with the goal of understanding the outcomes for TMVR in select patient populations. For patients with primary degenerative MR, the randomized controlled PRIMARY trial is an international, multi-center study assessing the long-term effectiveness and safety of transcatheter edge-to-edge repair compared with surgical repair over a ten-year period (Trial ID: NCT05051033). Primary outcomes for this trial will include all-cause mortality, need for MV reintervention, hospitalization rate for heart failure, and onset of ≥3+ MR. The CLASP IID/IIF Pivotal Clinical Trial demonstrated that the Edwards PASCAL Transcatheter Valve Repair System (Edwards Lifesciences, Irvine, California, United States) was noninferior to the Abbott MitraClip™ for major adverse events in MR patients that are prohibitive risk for surgery (Trial ID: NCT03706833). Multiple arms of the study will examine populations with either degenerative or functional MR. Comparative outcomes for this trial will include rates of major adverse events, MR severity reduction, and rate of heart failure hospitalization and/or mortality.

Many complexities exist in optimizing patient selection and selecting the best option for MV repair when surgical vs. TMVR options exist, especially for higher risk patients. The results from the aforementioned trials will be instrumental in helping to shape the role of TMVR for MR. Furthermore, tools to aid in patient-specific analysis of MR disease with computational modeling techniques may also prove to offer substantial guidance for the optimal MR intervention options.

## 3. Foundational work on computational modeling for TMVR

Computational modeling provides an avenue of exploration to cater individualized approaches to patients receiving TMVR/These simulations are derived from patients-specific clinical imaging and can provide a detailed understanding of the biomechanics of the ventricle and stress/strain on the mitral valve leaflets. Advances in imaging techniques now permit extremely high-fidelity computational models to be created from patient-specific anatomy. Computational modeling will be able to play an important role in predicting outcomes of TMVR (pre-intervention planning) and with assessment of valve geometry and function post-repair.

### 3.1. Finite element models

**Figure 1:**
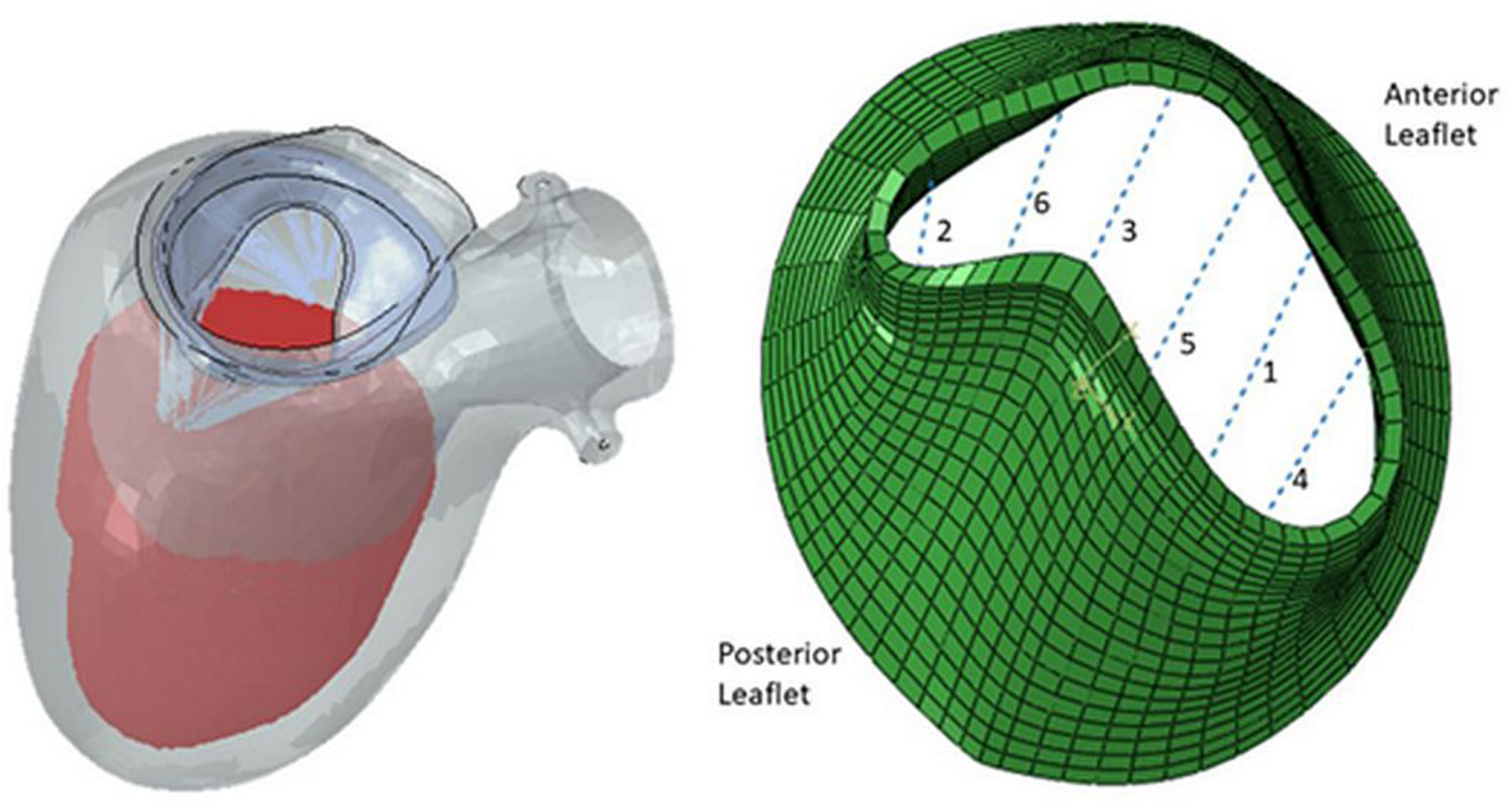
Finite element model of left ventricle and mitral valve apparatus (left) and mitral valve leaflets with six locations for possible MitraClip™ placement (right) (24).

There has been extensive study in MV modeling using finite element (FE) models to better understand the range of MV pathology. FE models, which are based upon solid geometry obtained from imaging can provide detailed information on the stress and strain within a mechanical system. FE models are comprised of three main elements: (1) geometry (anatomy obtained from clinical imaging,) (2) boundary conditions (pressures within chambers of the heart), and (3) material properties (behavior ventricle myocardium and the MV leaflets). A recent study utilized high-resolution magnetic resonance imaging to assess the mechanical effects of TMVR on leaflet stress and myocardial strain (Figure 1). While specific models can be generated to depict the MV or left ventricle alone, combined models of the mitral valve and left ventricle can now be more readily created, which offers greater degree of replicating the complex anatomic and physiologic relationship between them as the MV apparatus' papillary muscles are embedded within the left ventricle. One method of generating human FE models established by Ge et al. combined magnetic resonance imaging with trans-esophageal echocardiography (21). Three-dimensional echocardiographic images were used to outline the MV apparatus, while magnetic resonance imaging was used to capture geometry and strain of the left ventricle. A study by Zhang et al. featured FE models of functional MR using a sheep model (20). It was found that application of MitraClip™ increased both end-diastolic and end-systolic MV leaflet stress, leading to decreases in septo-lateral annular diameter and appreciable changes in procedure-related radial strain in the sub-valvular myocardium. Furthermore, these mechanical effects were compounded by enlarged left ventricles, as leaflet stress and procedure-related radial strain were increased during end-diastole in these models. It could be postulated that these biomechanical alterations from the clip may predispose to recurrent MR, though further studies with longer duration follow-up will be required.

**Figure 2:**
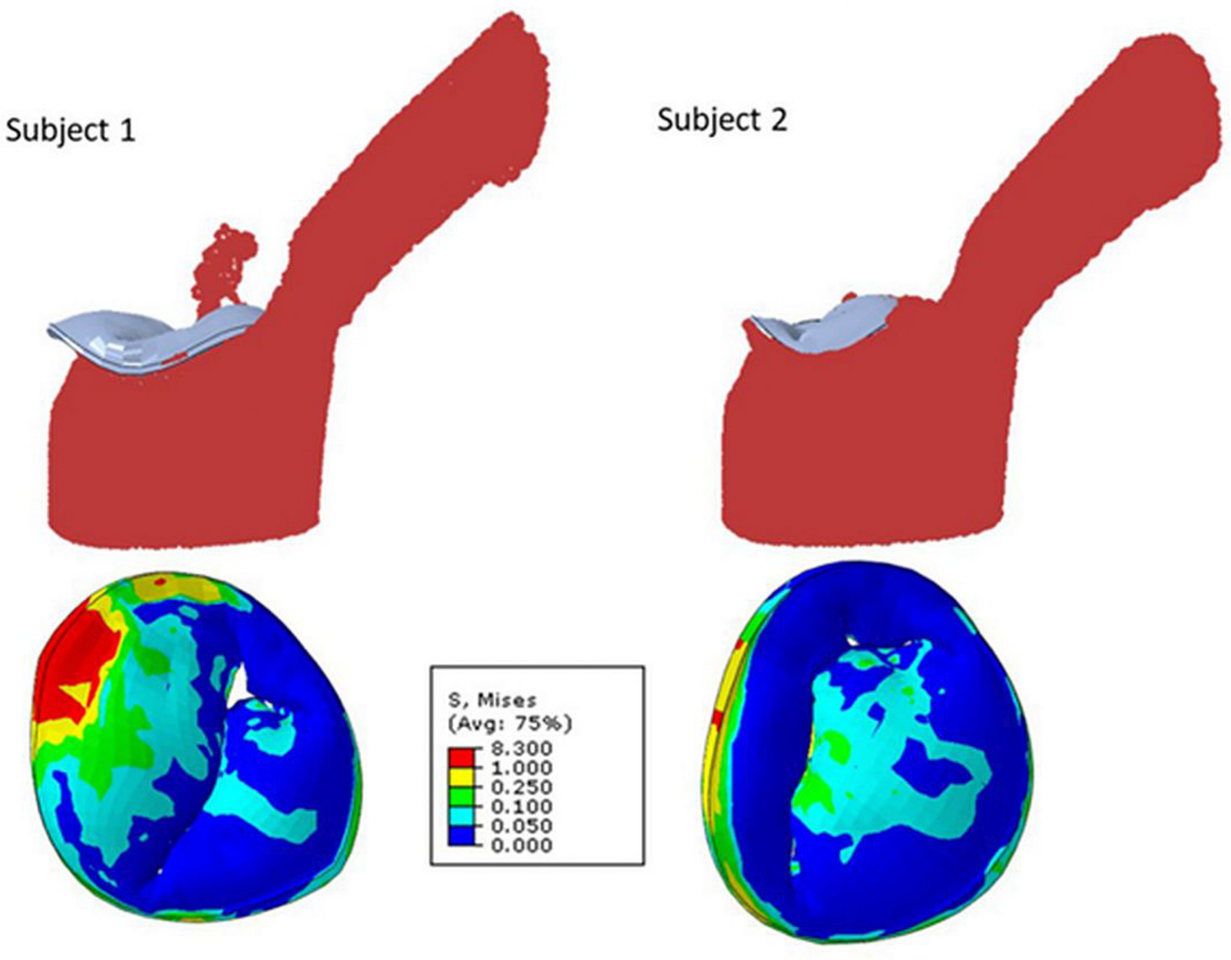
Mitral regurgitation models for two different subjects showing smooth particle hydrodynamics of regurgitation (upper row), and mitral valve leaflet von Mises stresses at systole (24).

### 3.2. Fluid structure interaction models

Fluid structure interaction models, which depict the interaction between deformable structures and its internal fluid flow, have been employed to imitate blood flow through the mitral valve and surrounding left ventricle and to quantitate reductions in MR following MitraClip™ placement (22). Blood flow was modeled using smoothed particle hydrodynamics, and the number of blood particles on the atrial side of the mitral valve was used to approximate MR. These fluid structural interaction models have described the effects of number and location of clips on reduction of MR. This methodology has similarly been used to assess the efficacy of clip implantation for tricuspid valve regurgitation (23).

One utilization of both finite element and fluid structure interaction models leverages an established atlas of shapes and complex scenarios to make clinical decisions on MitraClip™ implantation (24). To compose the atlas, mitral valve geometrical data was acquired from three different sources. Patients' 3D echocardiogram images were collected, and the pixel data from six key points from three views of the imaging were utilized. These coordinates were subsequently used to generate the geometry by morphing a template geometry. In addition, the dimensions of mitral valves from existing literature were used as reference for morphological data. Lastly, additional geometric images were created using principal component analysis and generative adversarial networks. From the acquired images, finite element (FE) software was used to simulate smoothed particle hydrodynamics of TMVR intervention in various scenarios. Results from these analyses of single- and multi-clip interventions demonstrated that the location and number of clips have effects on residual MR and extent of leaflet stress. However, FE models require hours to generate outcomes which may not be efficient in the clinical setting, and thus, the authors suggest that this atlas should be further explored with artificial intelligence models that can provide results in seconds.

## 4. Clinical utility of biomechanics and computational modeling for TMVR

In present clinical practice, location for MitraClip™ placement on the MV leaflets is primarily based on echocardiographic localization of the regurgitation jets. Advancement and greater translational use of computational modeling can help create new methods for pre-procedure clinical assessment. Computational modeling shows strong promise to play a role in shaping decision making algorithms surrounding surgical repair vs. TMVR, pre-procedural planning with the number and location of device placement in TMVR, and the creation of large atlases containing populations of MV geometries pre and post repair, linked to clinical outcomes, in order to optimize patient selection for TMVR vs. surgical repair.

Patient comorbidities play a significant role in determining whether to offer surgical MV repair vs. TMVR. Open surgical repair is associated with greater morbidity and mortality but can usually offer a durable, long-lasting repair. The Achilles heel of TMVR is the risk of incomplete treatment of MR and recurrent MR (17, 25, 26). While it may vary by institutional practice, in general, patients with acceptable surgical risk will be recommended for surgical MV repair, while patients with high or prohibitive risk for surgery will be advised to receive TMVR (27, 28). However, there are still questions about which approach should be offered to patients of intermediate risk. When having this pre-procedure discussion regarding surgical repair vs. TMVR, patient-specific models MV and left ventricle geometry can be generated to compare the most optimal outcome that could be afforded by each intervention. In particular, FE models can be constructed to provide detailed information on valve and myocardial stress. The surgical repair anatomy or clip configuration that would offer greatest reduction in MR, while minimizing regional peak stress on the valve apparatus and left ventricle can be simulated. This valuable information may help guide risk and benefits discussion surrounding the decision to recommend surgery or TMVR for certain patients.

Through larger scale studies of patient-specific MV geometries and intervention, we can now accrue populations of MR anatomy and intervention results termed an “atlas.” In addition to offering greater amounts of data on the biomechanics, the interventions and clinical outcomes, these atlases will help reduce the time needed to provide clinical answers to the team of cardiologists and cardiac surgeons evaluating an MR patient. Currently, a limitation of computational modeling technique in the clinical setting is the labor-intensive process required to generate accurate patient-specific models. Often, a single model may take up to 10–20 h of work by an experienced user, and may require several iterations of fine tuning to provide accurate results (19). However, progress is being made with the development of generic geometry model templates of the MV and left ventricle that can be rapidly morphed to match patient-specific anatomy.

We envision a clinical paradigm whereby the creation of the simulation of patient-specific geometry can be done with a semi-automated process directly from clinical imaging (echocardiography or cardiac computed tomography or magnetic resonance). As cases are accrued, the creation of an established atlas can allow a new patient-specific scenario to be matched to a similar or most-similar subset of cases to provide results of the outcome in an expedited manner. This could eliminate or reduce the need for de-novo simulations in many cases. Specific cases of rare anatomic variants or special circumstances that have few similar prior comparisons would prove to be the exception. This atlas will reduce the amount of time to obtain clinical meaningful results to help guide the cardiologists and cardiac surgeons on the decision to recommend TMVR or surgical repair, and would also hold valuable research potential.

## 5. Conclusion

Severe mitral regurgitation is a heart valve disease that leads to significant morbidity and mortality if left untreated. Current interventions include mitral valve repair through open or minimally invasive surgical repair or transcatheter mitral valve repair with technology such as the MitraClip™. Advances in computational modeling with the finite element method and fluid-structure interaction provides promising tools to facilitate patient selection for TMVR and for optimizing MR treatment outcomes. A large atlas of pathologies and biomechanical characteristics of MR scenarios can be established to help distinguish which patient populations should receive TMVR vs. surgical MV repair. Future clinical trials comparing outcomes using computational model aided decisions for procedural planning compared to current clinical practices for MR intervention will be needed. The present state of computational modeling techniques and the increasing ability to generate accurate patient-specific models position it to contribute significantly to our understanding of MR pathophysiology and optimal intervention strategies.

## References

[1] Douedi S Douedi H. Mitral regurgitation. StatPearls. (2022). https://www.ncbi.nlm.nih.gov/books/NBK553135/ (Accessed October 29, 2022). PMID: 31985928

[2] Apostolidou E Maslow AD Poppas A. Primary mitral valve regurgitation: update and review. Glob Cardiol Sci Pract. (2017) 2017:1–17. 10.21542/GCSP.2017.3 PMC6516795 31139637

[3] Wu S Chai A Arimie S Mehra A Clavijo L Matthews RV Incidence and treatment of severe primary mitral regurgitation in contemporary clinical practice. Cardiovasc Revasc Med. (2018) 19:960–3. 10.1016/J.CARREV.2018.07.021 30060923

[4] Iung B Baron G Butchart EG Delahaye F Gohlke-Bärwolf C Levang OW A prospective survey of patients with valvular heart disease in Europe: the euro heart survey on valvular heart disease. Eur Heart J. (2003) 24:1231–43. 10.1016/S0195-668X(03)00201-X 12831818

[5] Dal-Bianco JP Beaudoin J Handschumacher MD Levine RA. Basic mechanisms of mitral regurgitation. Can J Cardiol. (2014) 30:971–81. 10.1016/J.CJCA.2014.06.022 25151282 PMC4281524

[6] Seeburger J Katus HA Pleger ST Krumsdorf U Mohr F-W Bekeredjian R. Percutaneous and surgical treatment of mitral valve regurgitation. Dtsch Arztebl Int. (2011) 108:816. 10.3238/ARZTEBL.2011.0816 22211148 PMC3244168

[7] Sá MP Cavalcanti LRP Van den Eynde J Amabile A Escorel Neto AC Perazzo AM Respect versus resect approaches for mitral valve repair: a study-level meta-analysis. Trends Cardiovasc Med. (2022. 10.1016/J.TCM.2022.01.005 35051591

[8] Spiegelstein D Moshkovitz Y Sternik L Fienberg MS Kogan A Malachy A Midterm results of mitral valve repair: closed versus open annuloplasty ring. Ann Thorac Surg. (2010) 90:489–95. 10.1016/J.ATHORACSUR.2010.03.070 20667335

[9] Cetinkaya A Waheed M Bramlage K Liakopoulos OJ Zeriouh M Hein S Comparison of flexible, open with semi-rigid, closed annuloplasty-rings for mitral valve repair. J Cardiothorac Surg. (2021) 16:1–11. 10.1186/S13019-021-01405-1/FIGURES/5 33743744 PMC7981851

[10] Otto CM Nishimura RA Bonow RO Carabello BA Rwin JP Gentile F 2020 ACC/AHA guideline for the management of patients with valvular heart disease: a report of the American college of cardiology/American heart association joint committee on clinical practice guidelines. Circulation. (2021) 143:E72–E227. 10.1161/CIR.0000000000000923 33332150

[11] Colli A Adams D Fiocco A Pradegan N Longinotti L Nadali M Transapical NeoChord mitral valve repair. Ann Cardiothorac Surg. (2018) 7:812. 10.21037/ACS.2018.11.04 30598897 PMC6288219

[12] Colli A Manzan E Zucchetta F Bizzotto E Besola L Bagozzi L Transapical off-pump mitral valve repair with Neochord implantation: early clinical results. Int J Cardiol. (2016) 204:23–8. 10.1016/J.IJCARD.2015.11.131 26655529

[13] Alfieri O Maisano F De Bonis M Stefano PL Torracca L Oppizzi M The double-orifice technique in mitral valve repair: a simple solution for complex problems. J Thorac Cardiovasc Surg. (2001) 122:674–81. 10.1067/MTC.2001.117277 11581597

[14] Rey Meyer MA von Segesser LK Hurni M Stumpe F Eisa K Ruchat P. Long-term outcome after mitral valve repair: a risk factor analysis. Eur J Cardiothorac Surg. (2007) 32:301–7. 10.1016/J.EJCTS.2007.05.008 17561410

[15] Oh NA Kampaktsis PN Gallo M Guariento A Weixler V Staffa SJ An updated meta-analysis of MitraClip versus surgery for mitral regurgitation. Ann Cardiothorac Surg. (2021) 10:1–14. 10.21037/ACS-2020-MV-24 33575171 PMC7867427

[16] Vesely MR Benitez RM Robinson SW Collins JA Dawood MY Gammie JS. Surgical and transcatheter mitral valve repair for severe chronic mitral regurgitation: a review of clinical indications and patient assessment. J Am Heart Assoc. (2015) 4:2424. 10.1161/JAHA.115.002424 PMC4845273 26656862

[17] Feldman T Foster E Glower DD Kar S Rinaldi MJ Fail PS Percutaneous repair or surgery for mitral regurgitation. N Engl J Med. (2011) 364:1395–406. 10.1056/NEJMOA1009355/SUPPL_FILE/NEJMOA1009355_DISCLOSURES.PDF 21463154

[18] Swaans MJ Bakker ALM Alipour A Post MC Kelder JC De Kroon TL Survival of transcatheter mitral valve repair compared with surgical and conservative treatment in high-surgical-risk patients. JACC Cardiovasc Interv. (2014) 7:875–81. 10.1016/J.JCIN.2014.01.171 25147032

[19] Malik AH Zaid S Yandrapalli S Shetty S Aronow WS Ahmad H Trends and outcomes with transcatheter versus surgical mitral valve repair in patients ≥80 years of age. Am J Cardiol. (2020) 125:1083–7. 10.1016/j.amjcard.2019.12.050 31982103

[20] Zhang Y Wang VY Morgan AE Kim J Handschumacher MD Moskowitz CS Mechanical effects of MitraClip on leaflet stress and myocardial strain in functional mitral regurgitation—a finite element modeling study. PLoS One. (2019) 14:e0223472. 10.1371/JOURNAL.PONE.0223472 31600276 PMC6786765

[21] Ge L Morrel WG Ward A Mishra R Zhang Z Guccione JM Measurement of mitral leaflet and annular geometry and stress after repair of posterior leaflet prolapse: virtual repair using a patient specific finite element simulation. Ann Thorac Surg. (2014) 97:1496. 10.1016/J.ATHORACSUR.2013.12.036 24630767 PMC4121378

[22] Dabiri Y Mahadevan VS Guccione JM Kassab GS. A simulation study of the effects of number and location of MitraClips on mitral regurgitation. JACC: Advances. (2022) 1:100015. 10.1016/J.JACADV.2022.100015 PMC11198285 38939090

[23] Dabiri Y Yao J Sack KL Kassab GS Guccione JM. Tricuspid valve regurgitation decreases after mitraclip implantation: fluid structure interaction simulation. Mech Res Commun. (2019) 97:96–100. 10.1016/J.MECHRESCOM.2019.04.009 31439968 PMC6706066

[24] Dabiri Y Yao J Mahadevan VS Gruber D Arnaout R Gentzsch W Mitral valve atlas for artificial intelligence predictions of MitraClip intervention outcomes. Front Cardiovasc Med. (2021) 1738. 10.3389/FCVM.2021.759675 PMC8709129 34957251

[25] De Bonis M Lapenna E Buzzatti N La Canna G Denti P Pappalardo F Optimal results immediately after MitraClip therapy or surgical edge-to-edge repair for functional mitral regurgitation: are they really stable at 4 years? Eur J Cardiothorac Surg. (2016) 50:488–94. 10.1093/EJCTS/EZW093 27009105

[26] Sugiura A Kavsur R Spieker M Iliadis C Goto T Öztürk C Recurrent mitral regurgitation after MitraClip: predictive factors, morphology, and clinical implication. Circ Cardiovasc Interv. (2022) 15:E010895. 10.1161/CIRCINTERVENTIONS.121.010895 35193380

[27] von Ballmoos MCW Kalra A Reardon MJ. Complexities of transcatheter mitral valve replacement (TMVR) and why it is not transcatheter aortic valve replacement (TAVR). Ann Cardiothorac Surg. (2018) 7:724–30. 10.21037/ACS.2018.10.06 30598885 PMC6288216

[28] Ascione G Denti P. Transcatheter mitral valve replacement and thrombosis: a review. Front Cardiovasc Med. (2021) 401. 10.3389/FCVM.2021.621258 PMC8212998 34150861

